# Diverse and abundant viruses exploit conjugative plasmids

**DOI:** 10.1101/2023.03.19.532758

**Published:** 2023-04-21

**Authors:** Natalia Quinones-Olvera, Siân V. Owen, Lucy M. McCully, Maximillian G. Marin, Eleanor A. Rand, Alice C. Fan, Oluremi J. Martins Dosumu, Kay Paul, Cleotilde E. Sanchez Castaño, Rachel Petherbridge, Jillian S. Paull, Michael Baym

**Affiliations:** 1 Department of Biomedical Informatics, Harvard Medical School, Boston, MA 02115, USA; 2 Laboratory of Systems Pharmacology, Harvard Medical School, Boston, MA 02115, USA; 3 Department of Microbiology, Harvard Medical School, Boston, MA 02115, USA; 4 Boston University, Boston, MA 02215, USA; 5 Department of Systems Biology, Harvard Medical School, Boston, MA 02115, USA; 6 Roxbury Community College, Boston, MA, 02120, USA; 7 Broad Institute of MIT and Harvard, Cambridge, MA 02142, USA

## Abstract

Viruses exert profound evolutionary pressure on bacteria by interacting with receptors on the cell surface to initiate infection. While the majority of bacterial viruses, phages, use chromosomally-encoded cell surface structures as receptors, plasmid-dependent phages exploit plasmid-encoded conjugation proteins, making their host range dependent on horizontal transfer of the plasmid. Despite their unique biology and biotechnological significance, only a small number of plasmid-dependent phages have been characterized. Here we systematically search for new plasmid-dependent phages using a targeted discovery platform, and find that they are in fact common and abundant in nature, and vastly unexplored in terms of their genetic diversity. Plasmid-dependent tectiviruses have highly conserved genetic architecture but show profound differences in their host range which do not reflect bacterial phylogeny. Finally, we show that plasmid-dependent tectiviruses are missed by metaviromic analyses, showing the continued importance of culture-based phage discovery. Taken together, these results indicate plasmid-dependent phages play an unappreciated evolutionary role in constraining horizontal gene transfer.

Viral infections pose a constant threat to the majority of life on Earth^1,2^. Viruses recognize their hosts by interacting with structures (receptors) on the cell surface^3^. For viruses that infect bacteria (phages), these receptors are usually encoded on the chromosome, and are part of core cellular processes including transporter proteins or structurally integral lipopolysaccharides^4^. However, certain mobile genetic elements such as conjugative plasmids also contribute to the cell surface landscape by building secretory structures (e.g. conjugative pili) which enable them to transfer into neighboring bacterial cells^5,6^. Plasmid-dependent phages (PDPs) have evolved to use these plasmid-encoded structures as receptors, and can only infect plasmid-containing bacteria^7^. However, conjugative plasmids can transmit between distantly related cells, creating new phage-susceptible hosts by horizontal transfer of receptors^8^.

All previously known PDPs belong to unusual ‘non-tailed’ groups of phages, some of which have more in common with eukaryotic viruses than the ‘tailed’ phages that make up the majority of bacterial virus collections^9,10^. This includes the dsDNA alphatectiviruses, and members of the ssDNA inoviruses and +ssRNA fiersviruses. The handful of known PDPs have had profound impacts in molecular biology, enabling phage display technology^11^ (F plasmid-dependent phage M13), and *in vivo* RNA imaging^12^ (F plasmid-dependent phage MS2). PDPs have also aided in our understanding of the origin of viruses: tectiviruses are thought to represent ancient ancestors to adenoviruses^13^.

Predation by PDPs exerts strong selection on bacteria to lose conjugative plasmids or mutate/repress conjugation machinery such as the pilus^14–17^. As antibiotic resistance genes are frequently carried and spread by conjugative plasmids^18–22^, selection against plasmid carriage functionally selects against antibiotic resistance in many instances. The extent to which this is a significant evolutionary pressure on antibiotic resistance depends on how frequent these phages are in nature.

Despite the remarkable properties of these phages and their intriguing association with conjugative plasmids, only a handful of PDPs exist in culture. In the 1970s-80s at least 39 different PDPs were reported targeting 17 different plasmid types (classified by “incompatibility” groups)^7^. However, most of these reports predated the era of genome sequencing, and to our knowledge, most of the reported PDPs have been lost to science. Here we report that PDPs are not rare biological oddities, but rather a common, pervasive predator of conjugative plasmids. Using a targeted discovery assay, we find 64 new PDPs, dramatically expanding the known diversity of these phages. Moreover, we find that despite having been missed by metagenomic surveys, diverse PDPs are abundant and readily isolated from the environment.

## Co-culture enables direct discovery of plasmid-dependent phages

Almost all known plasmid-dependent phages were serendipitously identified by laborious retroactive screening of large phage collections that were isolated on bacteria with native conjugative plasmids^23^. In order to directly assess the abundance and diversity of PDPs in the environment, we set out to develop a targeted isolation approach. The challenge of targeted isolation is discriminating PDPs, in a direct, non-labor-intensive way, from other phages that depend on species-specific receptors (so-called “somatic” phages).

**Figure 1 |:**
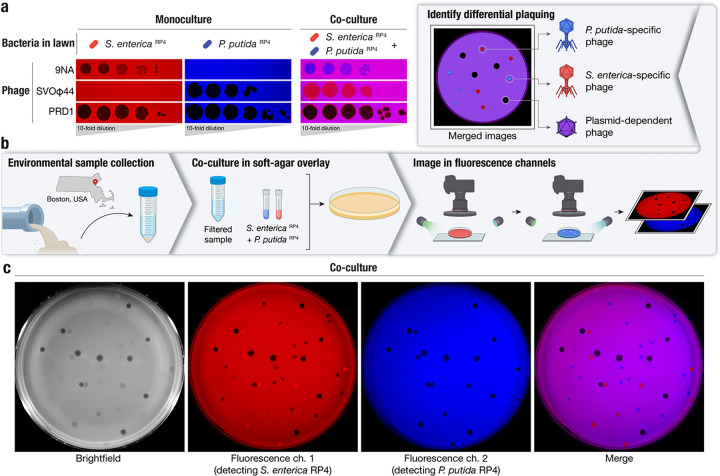
A method for systematic discovery of plasmid-dependent phages by fluorescence assisted co-culture (Phage DisCo) **a,** Comparison between monocultured lawns and a co-cultured lawn. All images show merged GFP and mScarlet fluorescence channels (GFP shown in blue for visualization purposes). In monocultured lawns with exclusively *S. enterica* ^RP4^ (red) or *P. putida* ^RP4^ (blue), only plasmid-dependent phage PRD1 and the appropriate species-specific phages (*S. enterica* phage 9NA or *P. putida* phage SVOϕ44) generate plaques. In the co-culture lawn (magenta, showing the overlap of both bacterial hosts), the species-specific phages form plaques on one of the species while plasmid-dependent phage PRD1 forms plaques on both species. **b,** Schematic of the Phage DisCo method and screening strategy. Environmental samples were collected from around Boston, USA, and processed into a co-culture lawn with two plasmid-carrying bacterial hosts labelled with different fluorescent markers. After incubation, the plates were imaged in both fluorescence channels. The merged image was then used to distinguish species specific phages (forming red or blue plaques) from plasmid-dependent phages (forming dark plaques) **c,** Imaging of co-cultured lawn with white light or fluorescent light channels, with approximately equimolar concentrations of phages shown in (b) to simulate a screening plate from an environmental sample containing plasmid-dependent and species-specific phages. Individual plaques are clearly discernible as 9NA (blue plaques), SVOϕ44 (red plaques), and PRD1 (dark plaques).

To differentiate PDPs, we co-cultured a pair of distinct bacteria sharing the same plasmid. As PDPs use the conjugative proteins produced by conjugative plasmids as receptors, their host range mirrors plasmid host range, and typically crosses bacterial genera. We selected a known PDP, the Alphatectivirus PRD1, which depends on IncP group conjugative plasmids and can infect the phylogenetically distant bacteria *Salmonella enterica* and *Pseudomonas putida* provided they contain an IncP plasmid, in this case RP4. We made a modification to the traditional phage plaque assay, by co-culturing these strains with differential fluorescent tags together in the same soft-agar lawn. After applying dilutions of phages, the phenotype of the PDP PRD1, which efficiently killed both fluorescently labeled strains on the lawn (resulting in no fluorescent signal) was immediately discernible from species-specific phage 9NA (infecting *S. enterica*) and SVOΦ44 (infecting *P. putida*) (Figure 1a). This observation formed the basis of the targeted phage discovery method we termed “Phage discovery by coculture” (Phage DisCo) (Figure 1b).

To directly isolate PDPs dependent on the RP4 plasmid using Phage DisCo, environmental samples containing putative PDPs can be mixed together with fluorescently labelled *S. enterica* and *P. putida* strains containing the conjugative plasmid RP4 (Figure 1b). After growth of the bacterial lawn, phages are immediately identifiable by the fluorescence phenotype of their plaques: *P. putida* phages appear as red plaques where only *S. enterica* RP4 (red) is able to grow, *S. enterica* phages present as blue plaques where only *P. putida* RP4 (blue) is able to grow, and PDPs make colorless plaques where both bacteria in the lawn are killed (Figure 1b). As a proof of principle, we mixed equimolar amounts of the test phages, 9NA, SVOΦ44 and PRD1, to simulate an environmental sample containing both species-specific phages and PDPs (Figure 1c). After incubation and growth of the bacteria in the lawn, the plate was photographed using a custom fluorescence imaging setup (Methods). Once the two fluorescent image channels were digitally merged, all three phages were easy to identify by fluorescence phenotype, and importantly, the PRD1 plaques could be easily discerned from the plaques made by the two species-specific phages.

## Plasmid-dependent tectiviruses from a limited geographic area fully encompass the previously known global diversity

Having established the efficacy of the method, we set out to look for PDPs in environmental samples using Phage DisCo. We chose to focus on phages depending on conjugative plasmids of the IncP incompatibility group, because only a handful of these phages have been described^7^, and they mostly belong to an unexplored family of lipid-containing phages, the Tectiviridae^24^. IncP plasmids are also associated with extensive antibiotic resistance gene cargo and are frequently isolated from environmental ^25^. The six known IncP-dependent tectiviruses (alphatectiviruses) are quite closely related despite being isolated from across the globe.

**Figure 2 |:**
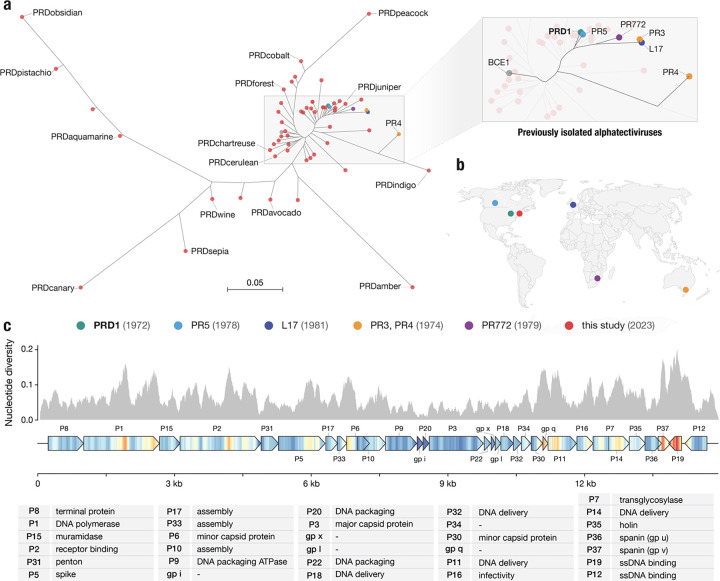
Targeted discovery of plasmid-dependent phages reveals unprecedented diversity and abundance **a,** Maximum likelihood tree of all known alphatectiviruses. (Generated with the whole genome, 14888 sites) Branch tips in red represent the novel phages isolated in this study. All other colors (highlighted in the enlarged section of the tree) represent all previously known representatives of this phage group. **b,** Map showing the site and isolation year of phages shown in (a). This collection includes and vastly expands the previously known diversity, despite being more geographically and temporally constrained **c,** Nucleotide diversity across our collection of alphatectivirus genomes (n=51). The genome map is colored to better display the nucleotide diversity value inside the gene body. Red coloration in the gene arrow symbols indicates high nucleotide diversity and blue indicates low nucleotide diversity, values correspond to the histogram above.

We discovered 51 novel plasmid-dependent phages (Figure 2a), using Phage DisCo with IncP plasmid RP4 to screen samples collected from compost, farm waste and wastewater in the Greater Boston area (Massachusetts, USA). All 51 discovered PDPs belong to the *Alphatectivirus* genus and are related to Enterobacteriophage PRD1. We adopted a naming system using the prefix “PRD” together with a color-based identifying name (e.g. PRDaquamarine, Supplementary Table S1). Surprisingly, despite our sampling being limited to a small geographical area and short time frame, the phages we isolated represented vastly more diversity than the six previously known plasmid-dependent tectiviruses that were isolated across multiple continents, suggesting these phages are greatly under sampled. We estimate our collection expands the genus *Alphatectivirus* from two species (PRD1 and PR4) to 12, as determined by pairwise nucleotide identity of all Alphatectiviruses, including the six previously known Alphatectiviruses and our 51 new isolates (Figure S1a) (species cut-off <95% nucleotide identity).

Additionally, by querying genome databases we identified one published tectivirus genome, *Burkholderia phage BCE1*, closely related to PRD1 by whole genome phylogeny (Figure 2b). As *Burkholderia sp.* are known hosts of IncP-type conjugative plasmids^26^ we expect that the *Burkholderia cenocepacia* host used to isolate BCE1 carried such a plasmid (highlighting the serendipitous nature by which PDPs are often found) and we include BCE1 in our known plasmid-dependent tectivirus phylogeny.

While the 51 new plasmid-dependent tectiviruses greatly expands the known diversity of this group of phages we found that all the phages in our collection had perfectly conserved gene synteny (Figure S1b). Just like the previously known alphatectiviruses, they have no accessory genome and contain all 31 predicted coding genes of the PRD1 reference genome, suggesting strong constraints on genomic expansion in this group of phages. However, they contain a large number of single nucleotide polymorphisms (SNPs) distributed across the genome (Figure 2c) and isolates ranged from 82.5% to 99% average pairwise nucleotide identity. Certain regions of the genome are highly associated with polymorphism, such as the center and C-terminus of the DNA polymerase gene. Two small genes, *XXXVII* and *XIX*, are especially associated with nucleotide polymorphisms across our genome collection. Interestingly, *XXXVII* (also called P37 or gp v) is the outer-membrane unit of a two-component spanin system thought to be responsible for fusion of the inner and outer membrane in the final stages of cell lysis^27^. Similarly, *XIX* is highly diverse across our collection, but its function as a ssDNA binding protein is redundant with the contiguous gene *XII* which is highly conserved. As plasmid-dependent tectiviruses are known to have a very broad host range dependent primarily on the presence of conjugative plasmids, we wondered whether the high diversity observed in specific genes might reflect specialization of some of the phages in our collection to infection of particular hosts.

## Plasmid-dependent tectiviruses show substantial phenotypic differences despite perfectly syntenic genomes with no accessory genes

**Figure 3 |:**
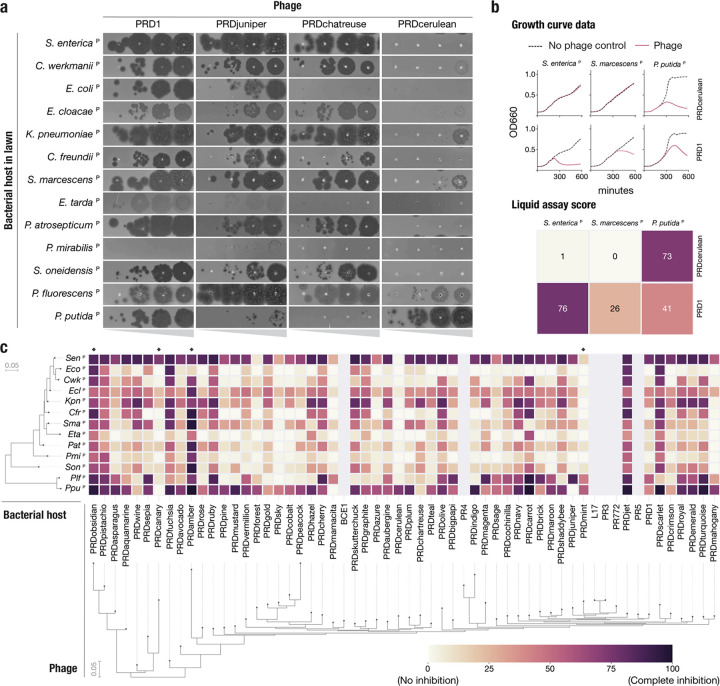
Plasmid-dependent tectiviruses have profoundly different host range preferences **a,** Plaque assays of 10-fold dilutions of five novel plasmid-dependent tectiviruses on diverse Gammaproteoabacterial hosts containing the IncP conjugative plasmid pKJK5 (indicated by ^P^). The five phages have large differences in plaquing efficiency on different host bacteria, despite being closely related by whole genome phylogeny (Figure 2a). **b,** Top shows examples of growth curve data for phages PRD1 and PRDcerulean on three host bacteria containing the pKJK5 plasmid. Bottom shows the same data, represented as liquid assay score. **c,** High throughput estimation of host range preferences for all the novel plasmid-dependent tectiviruses in our dataset by liquid growth curve analysis. Maximum likelihood trees at the left and bottom indicate the inferred phylogenetic relationships between phages (by whole genome phylogeny) and host bacteria (by 16S phylogeny). Grayed out rows are displayed for the 6 published alphatectiviruses that we were unable to collect host preference data for. Black diamonds on the top of the heatmap highlight phages with host range preferences that are referenced in the text.

Plasmid-dependent tectiviruses (alphatectiviruses) exhibit a remarkably wide host range^28^, surpassing the host breadth of any other described group of bacterial viruses. This ability comes in stark contrast with their small genome size, perfect gene synteny, and lack of accessory genome. To explore the extent to which this constrained genomic diversity leads to phenotypic variation in our collection of PDPs, we constructed a set of 13 hosts of diverse Gammaproteobacteria, carrying the IncP conjugative plasmid pKJK5 (indicated by ^P^). We initially observed that PDPs exhibited substantial differences in plaquing efficiency across hosts (Figure 3a). For example, while PRD1 is able to plaque efficiently in all but one of the hosts, PRDcerulean can only efficiently form plaques on two *Pseudomonas* hosts, representing a decrease in plaquing efficiency of at least four orders of magnitude in most other hosts. In contrast, PRDchartreuse and PRDjuniper decrease their plaquing efficiency by a similar magnitude in *P. putida*^P^ when compared against *P. fluorescens*^P^. Notably, these isolates share >95% nucleotide identity to PRD1 (Figure 2a, Figure S1a) and have no variation in gene content.

We quantified host preference differences of all 51 phages on all 13 bacterial species using a high throughput liquid growth assay^29^. For each phage-host pair we calculated a liquid assay score (see Methods), which represents the growth inhibition incurred by a fixed phage concentration, normalized as a percentage relative to the host growth in a phage-free control (Figure 3b,c). We found that, consistent with earlier plaque assays (Figure 3a), the growth inhibition phenotype was highly variable across phage isolates (Figure 3c). We identified more examples of phages such as PRDmint and PRDcanary that displayed a host-specialist behavior, akin to that of PRDcerulean, while others, like PRDobsidian and PRDamber appeared to robustly inhibit the growth of a wide range of hosts (host generalism). Surprisingly, when looking at the data broadly, we found that neither the phage nor the host phylogenetic relationships were strong predictors of host-preference. We speculate that these patterns might reflect the compositions of natural polymicrobial communities containing IncP plasmids, which require PDPs to rapidly adapt to infect particular assortments of taxonomically distant hosts.

## Metagenomic approaches fail to recover plasmid-dependent tectiviruses

**Figure 4 |:**
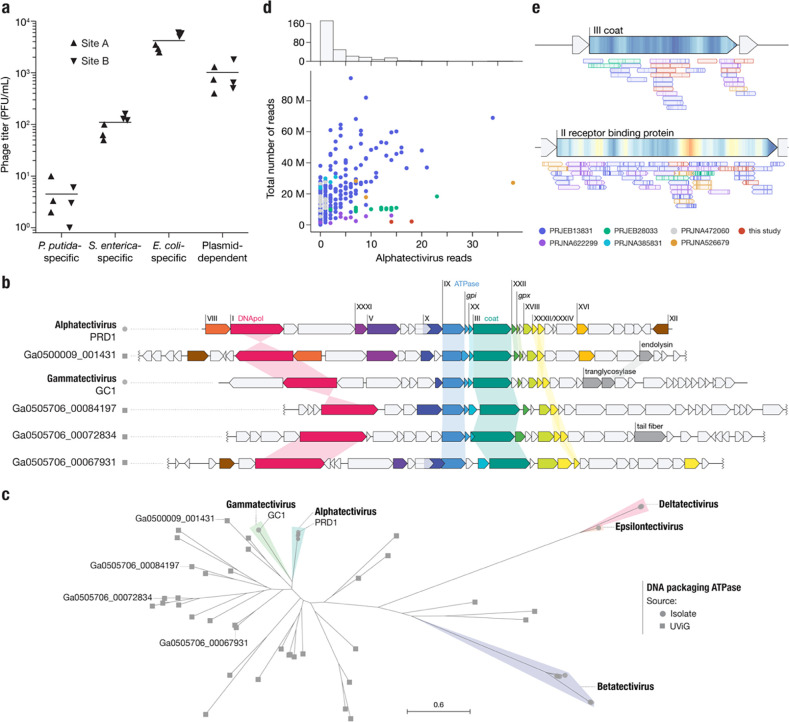
Alphatectiviruses are underrepresented in metagenomic assembled viromes **a,** Abundance of plasmid-dependent phages in wastewater influent. Plasmid-dependent phages targeting the IncP plasmid RP4 are orders of magnitude more abundant than *P. putida*- and *S. enterica*- specific phages, in two independent wastewater influent samples. **b,** Gene maps comparing Alphatectivirus PRD1 and Gammatectivirus GC1 against representative tectiviruses recovered from uncultivated viral genomes (UViGs). Colored genes represent homologs as detected by our protein models and shaded connectors represent proteins with >0.3 amino acid sequence identity. **c,** Maximum likelihood tree of the DNA packaging ATPase, including uncultivated tectiviruses (squares) and representatives of each genera of isolated tectiviruses (circles) **d,** Histogram and scatter plot of reads classified as being of alphatectiviral origin, against total number of reads in each metagenomic sample analyzed. Colors indicate different BioProjects from the SRA, full metadata can be found in Supplementary Table S1. **e,** Metagenomic reads mapped to regions of the PRD1 reference genome. ORFs are indicated with large arrows on top and colored as Figure 2d. Individual reads are represented by the smaller arrows and colored according to the dataset of origin (c), with mismatches marked as vertical lines.

Given the small number of plasmid-dependent tectiviruses known prior to this study (6, excluding BCE1) we were surprised by how easy it was to find these phages in our samples. However, a high absolute abundance of PDPs in hospital and municipal wastewater was recently reported in Denmark and Sweden^30^. To quantify their abundance, we used Phage DisCo to estimate the concentration of plasmid-dependent tectiviruses in fresh influent from two wastewater sites in Massachusetts, USA, relative to species-specific phages of *E. coli, S. enterica* and *P. putida* (Figure 4a). Phages dependent on the IncP plasmid RP4 were present in wastewater at approximately 1000 phages per mL, the same order of magnitude as species-specific phages of *E. coli* at ~4000 phages per mL. Species-specific phages of *S. enterica* and *P. putida* were less abundant than IncP-plasmid-dependent phages, present at ~100 phages per mL and ~5 phages per mL respectively. Wastewater is considered one of the best samples to find *E. coli* and *S. enterica* phages, and therefore the comparable levels of IncP-plasmid-dependent phages shows that these phages are common, at least in built environments (human-made environments). The extent to which this abundance is a characteristic of phages dependent on IncP type plasmids as opposed to PDPs in general remains to be seen.

Metagenomic-based viral discovery techniques have been extremely successful in expanding known viral diversity^31–33^. Although some studies have identified tectiviruses in metagenomic datasets^34^ and metagenomic-assembled genomes^35^, alphatectiviruses have yet to be found in metagenomic analyses, at odds with the relatively high abundance of the plasmid-dependent alphatectiviruses in wastewater (Figure 4a). With the increasing availability of metagenomic datasets, we aimed to reexamine the presence of this group of phages in assembled collections. We queried the JGI IMG/VR database of uncultivated viral genomes and retrieved genomes with a match to the Pfam model PF09018, which corresponds to the PRD1 coat protein. This search retrieved a set of diverse genomes in which, using refined models built from our alphatectivirus collection, we identified homology to diagnostic tectivirus genes^14^, such as DNA polymerase (*I*), ATPase (*IX*), and delivery genes (*XVIII*, *XXXII*) in addition to the coat protein (*III*) used for the retrieval of these sequences (Figure 4b). However, none of the uncultivated viral genomes appear to belong to any of the pre-existing groups of isolated tectiviruses (Figure 4c). This result highlights the large unexplored diversity of the Tectiviridae family.

We tested if we could recover alphatectivirus sequences through metagenomic sequencing of samples where we knew they were present at high abundance (around 1000 PFU/mL) (Figure 4a). We processed our samples by filtration, and further concentrated the viral fraction by 100-fold (Methods), before performing DNA extraction and bulk sequencing. We classified our metagenomics dataset with Kraken2 (See Methods) and found that a very small proportion of the reads (>0.001%) could be assigned to the alphatectivirus taxonomic group, which would not be sufficient for assembly (Figure 4d). This implied that, despite there being no assembled alphatectiviruses in public databases, they may still be identifiable in raw reads.

We then looked at additional published wastewater metagenomic sequencing datasets, and processed samples from diverse projects, representing different sequencing depths, locations, and sample processing methods, comprising a total of 290 samples and more than 5 billion reads total (Supplementary Table 1). Over 75% of the samples contained 5 or fewer reads assigned to alphatectiviruses. (Figure 4d) However, we found some alphatectivirus reads, primarily from the larger datasets, which directly mapped to the PRD1 reference genome (Figure 4e). The recovered reads appeared to be *bona fide* alphatectivirus sequences, as shown by the high mapping quality to the reference, a conservative approach that would fail to identify isolates with higher variation. Additionally, the diversity observed in the reads corresponds to the polymorphism landscape across our collection of isolates. For example, the coat protein, (gene III) is highly conserved across all tectiviruses and has very limited variation in the alphatectiviruses, while the receptor binding protein (gene II), a protein found exclusively in alphatectiviruses, has a much higher nucleotide diversity, reflected in the mapped metagenomic reads (Figure 4e). Taken together, no single dataset we analyzed contains enough reads to assemble a complete alphatectivirus genome. We hypothesize that a combination of a low relative abundance, small genome size, and highly polymorphic population might be responsible for the absence of alphatectiviruses in metagenomic assembled genome collections.

## Revisiting the F plasmid-dependent phage system uncovers a tailed plasmid-dependent phage

Given the successful isolation of novel IncP-dependent phage diversity with the Phage DisCo method, we next tested how generalizable the method was to other conjugative-plasmid systems. Given that IncF plasmid-dependent phages are the most well studied group of PDPs, we wondered if novel diversity remained to be discovered. All known phages dependent on IncF plasmid receptors can be classified into two groups; +ssRNA phages belonging to the Fiersviridae family (e.g. MS2 and Qbeta), and filamentous ssDNA Inoviridae (e.g. M13). The archetypal IncF plasmid, the F plasmid of *E. coli*, has a narrower host range than IncP plasmids, so we changed the coculture hosts strains to *E. coli* and *S. enterica*. As *S. enterica* strains natively encode an IncF plasmid, we used a derivative that had been cured of all plasmids and prophages to mitigate any interference from these elements.

**Figure 5 |:**
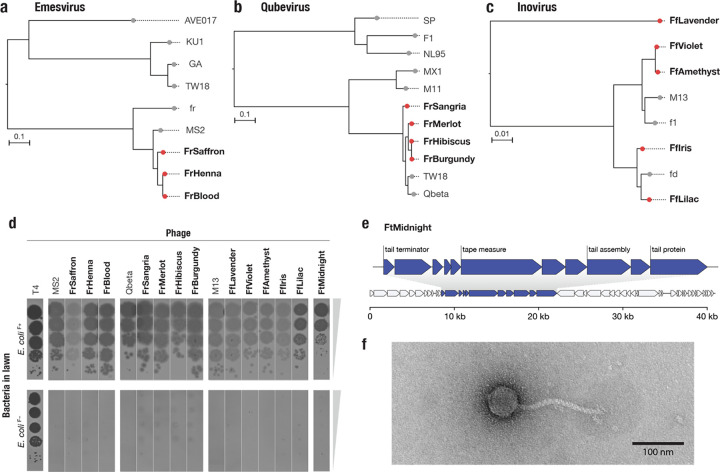
Phage DisCo uncovers new diversity even in the best-characterized (IncF) plasmid-dependent phage system **a,b,c,**Whole genome phylogenetic trees showing newly isolated (red) and known (gray) F dependent phages from the Emesvirus (RNA), Qubevirus (RNA) and Inovirus (ssDNA) genera. **d,** Confirmatory plaque assay of all newly isolated phages on *E. coli* host with and without the F plasmid, confirming plasmid dependency. **e,** Genome map of the novel IncF plasmid-dependent phage, FtMidnight, highlighting genes with predicted roles in tail formation in blue. **f,** Transmission electron micrograph of FtMidnight, confirming it has siphovirus morphology (long non-contractile tail).

In a limited screen we discovered 13 novel IncF PDPs (Figure 5). Three belonged to the Emesvirus genus and were closely related to MS2 (average of 92% nucleic acid identity to MS2) (Figure 5a, Figure S1c). Four of the phages were related to Qbeta (average of 94% identity to Qbeta), in the Qubevirus genus (Figure 5b, Figure S1c). Five of the IncF PDPs were novel Inoviruses related to M13 (Figure 5c, Figure S1c). Though we observed less diversity than in the IncP dependent phage screen, average nucleotide identity analysis suggests that one of the novel Inoviruses, FfLavender, represents a new phage species (FfLavender is 88% identical to M13 at the nucleic acid level). Like the IncP-dependent alphatectiviruses we isolated, all 12 of these new phages belonging to the Emesvirus, Qubevirus and Inoviruses genera had perfectly conserved gene synteny and no novel gene content relative to the closest reference phages (MS2, Qbeta and M13 respectively), suggesting that constrained genome content is a common feature of unrelated PDPs.

Finally, the final IncF plasmid-dependent phage we isolated, which we named FtMidnight, was found to have a dsDNA genome, clearly distinguishing it from any known IncF plasmid-dependent phage. Plaque assay confirmed that FtMidnight was dependent on the F plasmid (Figure 5d), and genome sequencing revealed it to be a 40,995 bp putatively tailed phage, indicated by the presence of numerous tail-associated genes (Figure 5e). Transmission electron microscopy confirmed that FtMidnight is a tailed phage resembling the morphological class of flexible tailed siphoviruses (Figure 5f). To our knowledge, FtMidnight is the first tailed phage found to depend on a conjugative-plasmid encoded receptor, demonstrating that novelty remains unsampled even in the well-studied F plasmid system.

## Discussion

Our finding that phages exploiting conjugative-plasmid encoded receptors are common and abundant in the urban environment suggests that PDPs potentially act as an important and previously unquantified constraint on the spread of conjugative plasmids in nature. With further study and discovery, PDPs could be exploited to manipulate the dynamics of conjugative plasmid mobility, and thus the spread of antibiotic resistance genes, in high-risk environments.

The relatively high abundance of IncP PDPs in wastewater measured by culture-based methods contrasts with their absence from metagenomic datasets, indicating a blind spot in bulk-sequencing based approaches to detect certain groups of viruses. The biochemical properties of some viruses have been suggested to play a role in their depletion from metagenomic datasets, such as DNA genomes with covalently bound proteins^36^. Though we cannot rule out a similar phenomenon is responsible for the lack of plasmid-dependent tectiviruses in metagenomic samples, we speculate that other factors might play a role, including the small genome size of PDPs relative to other viruses, low relative abundance, and high within sample sequence diversity interfering with consensus-assembly based methods. Still, the discrepancy points to the continued need for systematic culture-based viral discovery and method innovation.

Our discovery of FtMidnight, along with the significant expansion of known conjugative plasmid-dependent phage families, highlights the power of Phage DisCo to uncover unknown phage diversity. We anticipate this method to be generally applicable to identifying phages dependent on other conjugative plasmid systems, as well as translatable to further specialized phage discovery screens. The diversity and abundance of PDPs detected in the urban environment leads us to hypothesize that the interplay between phages and conjugative plasmids, both selfish genetic elements, may be driving the diversification of the conjugation systems mediating horizontal gene transfer in bacteria. This work represents a major first step in the exploration of this important group of phages, and much remains to be discovered about their ecology and biology, including how they interact with the plethora of defense systems present in bacteria^37^.

## Methods

### Strains and growth conditions

Details of all bacterial strains, plasmids, phages and primers used and constructed in this study are available in Supplementary Table S1. Unless stated otherwise, bacteria were grown at 37 °C or 30 °C in autoclaved LB^Lennox^ broth (LB: 10 g/L Bacto Tryptone, 5 g/L Bacto Yeast Extract, 5 g/L NaCl) with aeration (shaking 200 rpm) or on LB agar plates, solidified with 2% Bacto Agar at 37°C or 30 °C. Salt-free LBO media contained 10 g/L Bacto Tryptone, 5 g/L Bacto Yeast Extract. When required antibiotics were added at the following concentrations: 50 μg/mL kanamycin monosulfate (Km), 100 μg/mL ampicillin sodium (Ap), 20 μg/mL tetracycline hydrochloride (Tc), 30 μg/mL trimethoprim (Tm), 20 μg/mL chloramphenicol (Cm) and 20 μg/mL gentamicin sulfate (Gm).

### Phage replication

Replication host strains for all phages used in this study are detailed in Supplementary Table S1. High titer phage stocks were produced by adding ~10^5^ Plaque Forming Units (PFU) to exponential phase cultures at approximately OD_600_ 0.1, and infected cultures were incubated for at least 3 hours at 37 °C (with aeration). Phage lysates were spun down (10,000 X g, 1 min) and supernatants were filter-sterilized with 0.22 μm, syringe filters. Phage lysates were serial-diluted (decimal dilutions) with SM buffer and plaque forming unit (PFU) enumeration was performed by double-layer overlay plaque assay^38^, as follows. Bacterial lawns were prepared with stationary phase cultures of the host strains, diluted 40 times with warm top agar (0.5 % agar in LB, 55 °C). The seeded top agar was poured on LB 2% agar bottom layer: 3 mL for 8.6 cm diameter petri dishes or 5 mL for 8.6 cm × 12 cm rectangular petri dishes. When required, antibiotics were added to the top agar.

### Plasmid construction

The F plasmid from strain SVO150 was modified via recombineering to encode a *gfp* locus and kanamycin resistance locus (*aph*) for selection (FΔ*finO*::*aph*-P*lac*-*gfp*). Briefly, SVO150 was electroporated with the pSIM5tet recombineering plasmid (Supplementary Table S1), and the native IS3 interrupted *finO* locus was replaced with the *aph*-P*lac*-*gfp* cassette from pKJK5 using primers NQO2_9 and NQO2_12 as described (Koskiniemi et al., 2011). The replaced region was amplified with primers NQO2_5 and NQO2_6 and sent for Sanger sequencing to confirm the correct replacement.

### Strain construction

For differential identification of plaques in coculture and transconjugant selection, constitutive sgfp2* or *mScarlet-I* loci along with a chloramphenicol resistance locus were added to *E. coli*, *S. enterica* and *P. putida* strains (Supplementary Table S1). Tn7 transposons from pMRE-Tn7–145 and pMRE-Tn7–152 were introduced into the *att*^*tn7*^ site via conjugation from an auxotrophic *E. coli* donor strain as previously described^39^.

The RP4 plasmid was introduced into chromosomally tagged *S. enterica* and *P. putida* via conjugation using the BL103 donor strain. Overnight liquid cultures of donor and recipient strains were mixed at a 1:10 (donor:recipient) ratio and concentrated into a volume of 20 μl by centrifugation. The cell slurry was transferred to the top of a 12 mm, 0.45 μm nitrocellulose membrane on the surface of an LB agar plate for 4 hours at temperature optimal for the recipient strain (see Supplementary Table S1) to permit conjugation. Transconjugants were selected by plating on LB supplemented with chloramphenicol and kanamycin. For FΔ*finO*::*aph*-P*lac*-*gfp*, a plasmid and prophage-cured *S. enterica* strain (SNW555, D23580 ΔΦ ΔpSLT-BT ΔpBT1 ΔpBT2 ΔpBT3^40^) was used to mitigate any interference from the IncF *Salmonella* virulence plasmid (pSLT) and native prophages. The FΔ*finO*::*aph*-P*lac*-*gfp* plasmid was introduced into SNW55 and NQO62 via conjugation, exactly as described above.

For IncP-PDP host range experiments, the pKJK5 plasmid was transconjugated into *Pseudomonas putida* KT2440, *Pectobacterium atrosepticum* SCRI1043, *Shewanella oneidensis* MR1, *Serratia marcescens* ATCC 1388, *Enterobacter cloacae* ATCC 13047, *Pseudomonas fluorescens* Pf0–1, *Klebsiella pneumoniae* PCI 602, *Citrobacter werkmanii* IC19Y, *Citrobacter freundii* ATCC 8090, *Edwardsiella tarda* ATCC 15947, *Proteus mirabilis* BB2000 Δ*ugd* and *Salmonella enterica* serovar Typhimurium LT2 via the cross streak method. The pKJK5 plasmid contains *gfp* under the control of the P*lac* promoter, which results in derepressed fluorescence in non-*E. coli* (*lac* negative) hosts^41^. Additionally, the pKJK5 donor strain, NQO38, constitutively expresses *mCherry*, permitting easy identification of transconjugants without need for dual selection. Briefly, an overnight liquid culture of the donor strain NQO38 was applied vertically in a single streak down the center of an LB agar plate. Subsequently, an overnight liquid culture of a recipient strain was streaked horizontally across the plate, crossing over the donor streak. After incubation at the recipient optimal temperature, transconjugant colonies were purified on the basis of green fluorescence signal.

### Optimization of PDP detection by fluorescence-enabled co-culture

To validate the use of fluorescence-enabled co-culture to detect PDPs, a *S. enterica*-specific phage (9NA), a *P. putida-*specific phage (SVOΦ44) and an IncP plasmid-dependent phage were mixed at equal concentration (approximately 10^3^ PFU/mL). 100 μL each of overnight liquid cultures of *S. enterica* LT2 *att*^Tn7^::*Tn7-mScarlet-I* + RP4 (NQO89) and *P. putida att*^Tn7^::*Tn7*-SGFP2* + RP4 (NQO80) was added to 3 mL molten LB top agar, along with 10 μL of the phage mixture, and poured onto an LB agar plate. Plates were incubated overnight at 30 °C and then imaged in brightfield, red fluorescence channel, and green fluorescence channel using a custom imaging platform.

The custom imaging setup has a Canon EOS R camera with a Canon 100 mm lens with LEDs paired with excitation and emission filters (Green: 490–515 nm LED with 494 nm EX and 540/50 nm EM filters; Red: 567 nm LED with 562 nm EX and 641/75 nm EM filters). Excitation filters are held in a Starlight express emission filter wheel. The camera, LEDs, and filter wheel are all controlled with custom software. Exposure times were 0.25 [green] and 0.5 s [red], with camera set to ISO-200 and f/3.5 as experimentally determined to maximize dynamic range. Imagining parameters were selected such that when green and red fluorescence channel images were merged, all three phages could be easily identified by fluorescent plaque phenotype: 9NA phages were visible as green plaques (only *P. putida att*^Tn7^::Tn7-SGFP2* + RP4 grows in these areas), SVOΦ44 plaques were visible as red plaques (only *S. enterica* LT2 *att*^Tn7^::*Tn7-mScarlet-I* + RP4 grows in these areas) and PRD1 plaques had no fluorescent signal (neither species grew in these areas). The red and green channels were separated from their raw images, their exposure linearly rescaled, and remapped to the red and blue channels respectively (to enhance visual color contrast). All image manipulations were done with scikit-image v0.17.2 ^42^.

### Collection and processing of environmental samples

For phage isolation, wastewater primary influent from a total of 4 sites in Massachusetts were collected, along with soil, animal waste, and compost from farms, community gardens and parks close to Boston, USA. All samples were resuspended (if predominantly solid matter) in up to 25 mL of sterile water and incubated at 4 °C for 12 hours with frequent vortexing to encourage suspension and homogenization of viral particles. The resuspended samples were centrifuged at 4,000 × g for 30 minutes to pellet large biomass, and the clarified supernatant was filter sterilized using a 0.22 μm vacuum driven filtration unit to remove bacteria. Filtered samples were stored at 4 °C. For metaviromic sequencing and phage enumeration in wastewater influent, two 100 mL samples were collected in September 2022 from two separate intake sources of wastewater at a treatment plant in Boston, MA. Samples were processed by filtration as described above, except that processing was initiated immediately upon sample collection to avoid any sample degradation.

### Isolation of novel environmental PDPs by fluorescence enabled coculture

For high throughput discovery of plasmid-dependent phages targeting the IncP plasmid pilus, co-culture lawns of *S. enterica* LT2 *att*^Tn7^::*Tn7-mScarlet-I* + RP4 (NQO 89) and *P. putida attTn7*::Tn7-SGFP2* + RP4 (NQO80) were prepared as described earlier, except that 100 μl of filtered environmental samples putative novel phages were added instead of the reference phages. In cases where phage load in samples was too high, and subsequent lawn did not grow uniformly due to widespread lysis, the amount of filtered sample added to the lawns was diluted 10-fold until single plaques were obtained. Putative PDP plaques (exhibiting no fluorescence) were sampled using sterile filter tips, diluted and replated for single plaques at least twice to ensure purity. For the IncF plasmid targeting phages, the procedure was the same, except that strains SVO348 (*E. coli* MG1655 *att*^Tn7^*::mScatlet-I-gmR* + FΔ*finO*::*aph-gfp*) and NQO87 (*S. enterica* D23580 ΔΦ ΔpSLT-BT ΔpBT1 ΔpBT2 ΔpBT3+ FΔ*finO::aph-gfp*) were used in the lawns. The plasmid and prophage cured strain of *S. enterica* was used for the IncF-dependent phage screen to mitigate interference from the native *Salmonella* virulence plasmid (which belongs to incompatibility group F^43^) and prophages.

Once putative novel PDPs had been purified from environmental samples, 5 μl drops of 10-fold dilutions were plated on lawns of isogenic plasmid free host strains (BL131, SVO126, SVO50 or SNW555) to confirm plasmid-dependency. We note that false positives (i.e plasmid independent phages that infected both species in the coculture) were occasionally obtained during the IncF PDP isolation, due to the phylogenetic proximity between *E. coli* and *S. enterica*, suggesting that use of more distinct host strains (if possible for the plasmid of interest) maximizes assay efficiency.

### Phage DNA and RNA extraction and sequencing

Pure phage stocks that had undergone at least 2 rounds of purification from single plaques and had titers of at least 10^9^ PFU/mL were used for nucleic acid extraction. The Invitrogen Purelink viral RNA/DNA mini kit was used to extract genetic material from all phages according to manufacturer instructions. High absorbance ratios (260/280) 2.0–2.2 were considered indicative of RNA phage genomes. To remove host material contamination, putative RNA samples were incubated with DNase I (NEB) for 1 hour at 37 °C and inactivated afterwards with EDTA at a final concentration of 5 mM. RNA was reverse transcribed using SuperScript^™^ IV VILO^™^ (Invitrogen^™^) for first strand synthesis, per the manufacturer’s instructions. Second strand synthesis was performed by incubating the cDNA with DNA Ligase, DNA Polymerase I, and RNase H in NEBNext^®^ Second Strand Synthesis Reaction Buffer (NEB) at 16 °C for three hours. cDNA was then used in downstream library preparation. Additionally, as all known non-RNA IncF plasmid-dependent phages have ssDNA genomes which are incompatible with tagmentation-based library preparation, any putative DNA sample from IncF plasmid-dependent phages was subjected to second strand synthesis as described above. Illumina sequencing libraries of the DNA and cDNA samples were prepared as previously described^44^. Sequencing was carried out on the Illumina Novaseq or iSeq with 150 bp paired end cycles. The genetic composition (dsDNA vs ssDNA) for phage FtMidnight was inferred via fluorescence signal using the Quant-IT dsDNA kit (Invitrogen).

For metaviromic DNA extraction, 45 mL of freshly filtered influent from each of the two extraction sites was concentrated 100 X into 500 μl using 100 kDa molecular weight cut off centrifugal filter units (Amicon). Nucleic acids were extracted from 200 μl of concentrated filtrate, and sent to SeqCenter for library preparation and Illumina sequencing. Sample libraries were prepared using the Illumina DNA Prep kit and IDT 10 bp UDI indices, and sequenced on an Illumina NextSeq 2000, producing 2×151 bp reads.

### Phage genome assembly and annotation

Sequencing reads were adapter trimmed (NexteraPE adapters) and quality filtered with Trimmomatic v.0.39^45^. For samples with very high read depth, filtered reads were subsampled with rasusa v.0.5.0^46^ to an approximate 200x coverage to facilitate assembly. The reads were then assembled with Unicycler v.0.4.8 ^47^. The annotations from curated PRD1, MS2, Qbeta and M13 reference genomes were transferred to the resulting assemblies with RATT v.1.0.3^48^ and manually curated for completion. Phage isolates with redundant genomes were removed from the analysis and all phages included in this study represent unique isolates. Reads are deposited in the NCBI Sequence Read Archive (SRA) (accessions pending) All accession numbers for previously published genomes and those generated in this study are listed in Supplementary Table S1.

### Nucleotide diversity

To calculate nucleotide diversity among the alphatectiviruses, all the assembled isolates were aligned to the PRD1 reference genome with minimap2 v2.24^49^. Resulting alignments were processed with bcftools v1.9^50^ and samtools v1.6^51^ to then calculate nucleotide diversity with vcftools v0.1.16^52^ with a sliding window of size 100 bp. Results were plotted with seaborn v0.12.2^53^ and matplotlib^54^. Novel species classifications were proposed where average pairwise nucleotide diversity was less than 95%^55^.

### Phage enumeration in wastewater by plaque assay

Two freshly filtered wastewater influent samples were processed as previously described (See Collection and processing of environmental samples) and the concentration of phages in volumes of 10, 100 and 500 μm were enumerated by single host plaque assay on strains SVO50, BL131, and SVO126 and by fluorescence enabled co-culture plaque assay on NQO89 and NQO80. All phage enumeration was performed with 3 biological replicates. Titers per mL were calculated and plotted for both sites.

### Determination of phage host range

Host range of the IncP-PDPs was assessed by traditional efficiency of plating (EoP) assay or by killing in liquid culture by OD_660_ measurement, based on a previously described method^29^. All the phages were challenged against the following bacteria containing the pKJK5 plasmid: *Pseudomonas putida* KT2440*, Pectobacterium atrosepticum* SCRI1043*, Shewanella oneidensis* MR1, *Serratia marcescens* ATCC 1388, *Enterobacter cloacae* ATCC 13047, *Pseudomonas fluorescens* Pf0–1, *Klebsiella pneumoniae* PCI 602, *Citrobacter werkmanii* IC19Y, *Citrobacter freundii* ATCC 8090, *Edwardsiella tarda* ATCC 15947, *Proteus mirabilis* BB2000 Δ*ugd* and *Salmonella enterica* serovar Typhimurium LT2. These hosts were chosen as they all showed some degree of susceptibility to IncP dependent phages when transconjugated with the pKJK5 plasmid, indicating proper elaboration of the IncP pilus.

For the high throughput determination of host range, phages were normalized to a titer of 10^7^ PFU/mL as measured in strain NQO36, with the exception of PRDchartreuse, PRDcanary, PRDjuniper, and PRDmamacita, which were normalized to the same titer in NQO37, due to their inability to replicate to high titers in NQO36. Growth curve experiments were set up in 96-well plates with each well containing 180 μL of bacterial culture at OD600 of ~0.1 and 20 μL of phage stock when appropriate, for a final concentration of 10^6^ PFU/mL. They were grown in a plate reader (Tecan Sunrise^™^) for 10 hours with shaking, at the optimal temperature for the strain (see Supplementary Table S1), measuring the optical density at 660nm, every 5 minutes. Each 96-well plate had a phage-free control, cell free control, and the strain-phage condition in triplicate. To calculate the liquid assay score of each host-phage pair we followed the method described previously^29^. Briefly, we calculate the area under the growth curve for each host-phage pair, as well as for its corresponding phage-free control grown in the same plate. The mean area under the curve value is then normalized as a percentage of the mean area under the curve in the phage-free control. Growth curves are plotted with shading representing the standard error. Liquid assays scores are plotted as a heatmap, and are vertically sorted according to the previously computed alphatectivirus tree and horizontally sorted according to a 16S tree of the bacterial hosts (See Supplementary Table S1)

### Search and comparison of tectiviruses in metagenomic assembled genomes

To collect metagenomic assembled genomes of tectiviruses, a search was performed in the JGI IMG/VR^56^ for uncultivated viral genomes (UViGs) matching Pfam model PF09018^57^, which corresponds to the tectivirus capsid protein. The recovered assemblies were annotated with prokka v1.14.6^58^ using the PHROGs database^59^. To refine these annotations, our large collection of alphatectiviruses was used to build protein alignments for each protein in the PRD1 genome, using clustalo v1.2.4.^60^ and manually curating them for quality. These alignments were then used to build hmm profile models with HMMER v3.3.1^61^, to search them against the collected tectivirus MAGs. A representative selection of annotated MAGs was selected and visualized with clinker v0.0.27^62^ and colored to show homology. Shaded connectors represent proteins with >0.3 sequence identity, while annotations with the same color represent significant (p < 0.01) homologs according to the HMMER search.

### Search for alphatectiviruses in metagenomic reads

Kraken2 v2.1.2^63^ was used to search for the presence of alphatectiviruses reads in metagenomic datasets. A custom database was built by adding our new alphatectivirus assemblies to the default RefSeq viral reference library. With this database, a collection of reads from wastewater sequencing projects was searched. The SRA BioProject accession numbers and metadata of this collection can be found in Supplementary Table S1. The individual reads from each sequencing run that were classified as belonging to alphatectiviruses according to Kraken2 were extracted and mapped to the PRD1 reference genome with minimap2 v2.22. The resulting mapped reads were processed with samtools v1.6 and visualized with IGV v2.11.4^64^.

### Phylogenetic trees

For the Alphatectivirus, Emesvirus, Qubevirus, and Inoviridae trees, previously published genomes and those collected in this study were aligned with clustalo v1.2.4. The resulting multiple sequence alignment was manually curated to ensure quality of the alignment. Trees were then built with iqtree v2.2.0.3^65^ and phyml v3.2.0.^66^, and visualized with iTOL v6.7^67^. For the tectivirus ATPase tree, the amino acid sequences for protein P9 (ATPase) from all known tectiviruses were aligned with clustalo v1.2.4. This alignment was used to create an hmm profile model with HMMER, which was then used to search the amino acid sequences extracted from the annotated MAGs (see Search for tectiviruses in metagenomic assembled genomes). Significant hits were extracted and aligned to the model with HMMER. We also included in this alignment the previously metagenomic-assembled tectiviruses listed in Yutin et al.^35^ and a selection of characterized representatives of the 5 tectivirus genera. A tree of the resulting ATPase alignment was built with phyml v3.2.0, and visualized with iTOL v6.7. All accession numbers of sequences used to build this tree are listed in Supplementary Table S1.

### Electron microscopy

Carbon grids were glow discharged using a EMS100x Glow Discharge Unit for 30 seconds at 25mA. High titer phage stocks were diluted 1:10 in water and 5 μL was adsorbed to the glow discharged carbon grid for 1 minute. Excess sample was blotted with filter paper and the grids were washed once with water before staining with 1% uranyl acetate for 20 seconds. Excess stain was blotted with filter paper and the grids were air dried prior to examination with a Tecnai G2 Spirit BioTWIN Transmission Electron Microscope at the Harvard Medical School Electron Microscopy Facility.

## Supplementary Material

Supplement 1 Figure S1. a, Whole-genome pairwise nucleotide identity matrix comparing all known alphatectiviruses. Highlighted isolates represent proposed new species. b, Gene map comparison of all alphatectiviruses I solated in this study, colors are as in (Figure 4b) c, Whole genome assemblies of IncF-dependent phages aligned to their corresponding reference genome. Grey area represents an aligned region, vertical lines represent SNPs relative to the reference, and capped vertical lines represent INDELs. Incomplete ends of the assembly are represented by horizontal lines.

Supplement 2 Table S1. Excel spreadsheet listing all bacterial strains, plasmids, phages, primers and SRA datasets used in this study

## Data Availability

Raw sequencing reads have been deposited in the NCBI BioProject database under accession number PRJNA954020. Accession numbers for novel phage genomes generated in this study can be found in Supplementary Table S1. All raw data used in figures are available on a github repository: https://github.com/baymlab/2023_QuinonesOlvera-Owen All unique materials used are available from the authors on request.
