# Supplementary material for: Diverse and abundant viruses exploit conjugative plasmids
Source: bioRxiv. 2023 Apr 21:2023.03.19.532758. Originally published 2023 Mar 19. Preprint. [Version 2] doi: 10.1101/2023.03.19.532758 (PMC10055259; doi:10.1101/2023.03.19.532758)
Supplement: Supplementary file 1 [file media-1.pdf]

Supplementary Figures

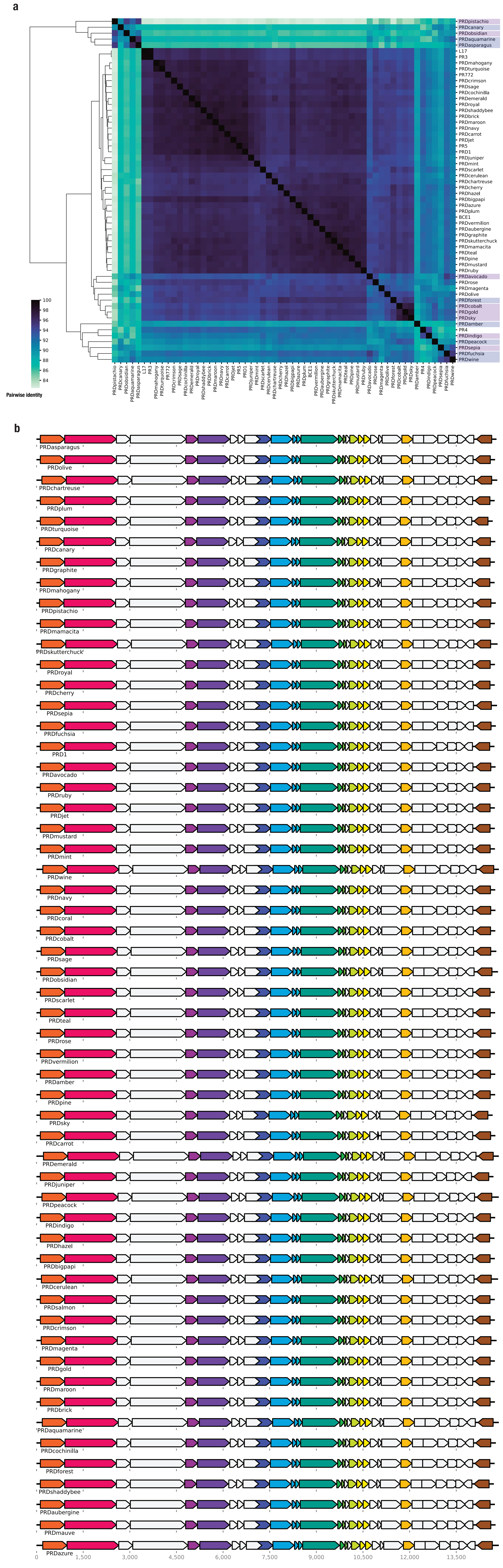

Figure S1 | Alphatectiviruses have no variation in gene content, but contain a large number of single nucleotide polymorphisms. a, Whole-genome pairwise nucleotide identity matrix comparing all known alphatectiviruses. Highlighted isolates represent proposed new species. **b,** Gene map comparison of all alphatectiviruses isolated in this study, colors are as in (Figure 4b)
